# A nomogram for individually predicting overall survival for elderly patients with early breast cancer: a consecutive cohort study

**DOI:** 10.3389/fonc.2023.1189551

**Published:** 2023-07-28

**Authors:** Ying Zhong, Yidong Zhou, Yali Xu, Zhe Wang, Feng Mao, Songjie Shen, Yan Lin, Qiang Sun, Kai Sun

**Affiliations:** ^1^ Department of Breast Disease, Peking Union Medical College Hospital, Beijing, China; ^2^ Medical Research Center, State Key Laboratory of Complex Severe and Rare Diseases, Peking Union Medical College Hospital, Chinese Academy of Medical Sciences and Peking Union Medical College, Beijing, China

**Keywords:** breast cancer, elderly patients, predictive nomogram, overall survival, comorbidities

## Abstract

**Background:**

Elderly patients with breast cancer are highly heterogeneous, and tumor load and comorbidities affect patient prognosis. Prediction models can help clinicians to implement tailored treatment plans for elderly patients with breast cancer. This study aimed to establish a prediction model for breast cancer, including comorbidities and tumor characteristics, in elderly patients with breast cancer.

**Methods:**

All patients were ≥65 years old and admitted to the Peking Union Medical College Hospital. The clinical and pathological characteristics, recurrence, and death were observed. Overall survival (OS) was analyzed using the Kaplan–Meier curve and a prediction model was constructed using Cox proportional hazards model regression. The discriminative ability and calibration of the nomograms for predicting OS were tested using concordance (C)-statistics and calibration plots. Clinical utility was demonstrated using decision curve analysis (DCA).

**Results:**

Based on 2,231 patients, the 5- and 10-year OS was 91.3% and 78.4%, respectively. We constructed an OS prediction nomogram for elderly patients with early breast cancer (PEEBC). The C-index for OS in PEEBC in the training and validation cohorts was 0.798 and 0.793, respectively. Calibration of the nomogram revealed a good predictive capability, as indicated by the calibration plot. DCA demonstrated that our model is clinically useful.

**Conclusion:**

The nomogram accurately predicted the 3-year, 5-year, and 10-year OS in elderly patients with early breast cancer.

## Background

The incidence of breast cancer increases with age, and patients with breast cancer mainly comprise the elderly population (1, 2). Breast cancer-related mortality declines with age (3). The proportion of pathological types with good prognosis, such as luminal-type cancers, gradually increases with age (4). Many elderly patients with breast cancer die from age-related comorbidities rather than breast cancer (5). The survival rate in elderly patients with complications after breast cancer surgery is lower than that in patients without complications (6). Older patients are underrepresented in most clinical trials and few clinical studies have focused on older patients with breast cancer (7–10). Therefore, the risks and benefits of anti-cancer therapy should be carefully evaluated in older patients with breast cancer (11). Owing to the heterogeneity of elderly patients with breast cancer, a predictive model is needed to guide clinicians in making specific recommendations for these patients.

Adjuvant! Online is the most widely used prediction tool for breast cancer (12) and inaccurately predicts overall survival (OS) and recurrence in elderly patients with breast cancer (13). The PREDICT tool can predict the 5-year OS in elderly patients with breast cancer, but it does not take into account comorbidities (14). G8 and modified-G8 screening tools were identified as strong predictors of OS in older patients with cancer. However, they do not target patients with breast cancer because the G8 items do not evaluate tumor characteristics (15).

The aim of this study was to construct and validate a nomogram by studying its predictions for 3-year, 5-year, and 10-year OS based on a consecutive cohort of elderly patients with breast cancer at Peking Union Medical College Hospital (PUMCH).

## Materials and methods

### Study population

In this study, patients were recruited from a cohort of elderly patients with early breast cancer at the PUMCH. All consecutive patients were ≥65 years of age and underwent breast cancer surgery at PUMCH between 2000 and 2020. Demographic information, comorbidities, clinical characteristics, surgical method, and chemotherapy, endocrine therapy, and radiotherapy regimens were collected after the patients were enrolled (16, 17). Follow-ups were conducted via outpatient or telephone interviews. The final follow-up was conducted on 30 June 2022. The median follow-up period was 54 months (6–190 months). In this study, all patients underwent surgery (either lumpectomy or mastectomy with axillary lymph node staging or sentinel lymph node biopsy). All specific comorbidities, including hypertension, diabetes, and myocardial ischemia, were recorded to evaluate the Charlson comorbidity index (CCI).

All pathological tests were performed at the Department of Pathology at PUMCH. Ki67 status was divided into high and low groups (14% cutoff). Human epidermal growth factor receptor 2 (HER2) status was defined as negative with 0 or 1+ on immunohistochemistry (IHC), positive with 3+ on IHC or positive on FISH staining, and unknown (UK) with 2+ on IHC or without FISH staining. Patients with stage N0 disease were defined as those without suspected clinical axillary lymph nodes who did not undergo axillary surgery or those who underwent axillary surgery and had negative pathological lymph nodes. Patients with N1, N2, and N3 tumors were defined as 1–3 positive pathological axillary lymph nodes, 4–9 positive pathological axillary lymph nodes, and ≥10 positive pathological axillary lymph nodes, respectively, after axillary surgery. Patients who underwent different operation types were divided into three main groups: (1) patients who underwent lumpectomy or mastectomy without axillary surgery, (2) patients who underwent lumpectomy and sentinel lymph node biopsy or axillary lymph node dissection, and (3) patients who underwent mastectomy and sentinel lymph node biopsy or axillary lymph node dissection.

Comorbidities were stratified according to the CCI (18). The age-adjusted CCI (ACCI) score has been used to predict survival in different kinds of disease (19, 20) and various types of cancer (21, 22). Each patient in our cohort was calculated a comorbidity score excluding age according to ACCI (https://www.mdcalc.com/charlson-comorbidity-index-cci). The patients with scores of 0, 1, 2, and ≥3 were classified as the CCI (0), CCI (1), CCI (2), and CCI (3) groups, respectively.

### Statistical analysis

Continuous data are presented as mean ± standard deviation or median [interquartile range (IQR), 25%–75%] according to data distribution. Categorical data are expressed as numbers (*n*) and percentages (%). Continuous variables with normal distribution between the training and validation cohorts were compared using Student’s *t*-test, and those with abnormal distribution were compared using the Mann–Whitney *U* test. Pearson’s chi-squared test was used for categorical variables. The distribution and patterns of missing data were assessed. Multiple imputations were performed using the Random Forest Algorithm because the predictor variables were assumed to be missing at random.

Survival analyses were performed using the Kaplan–Meier log-rank test. Cox proportional hazards regression was used to assess the association between predictor variables and OS, expressed as hazard ratios (HRs) with 95% confidence intervals (CIs). Predictor variables associated with overall mortality in the univariate analysis were further examined in a multivariable Cox proportional hazards regression, which was tested using the scaled Schoenfeld residuals method. A global *p*-value greater than 0.05 indicates that the whole model conformed to the PH hypothesis. We used stepwise selection to identify the most significant variables for inclusion in the model. Akaike’s information criterion (AIC) and −2 log-likelihood ratio (−2LLR) algorithm were used to identify the best predictor variables of the final model that predicted overall death in the training cohort. We also evaluated the interactions between selected variables. The variance inflation factor (VIF) was assessed among the covariates in the Cox model, and VIF > 4.0 was interpreted as indicating multicollinearity.

A nomogram was constructed based on Cox proportional hazards regression for 3/5/10-year OS probability. The discrimination and accuracy of the nomogram were assessed in both the training and validation cohorts. Discrimination was measured by the bias-corrected Concordance index (C-index) with bootstrap method. Accuracy was assessed by calibration plots using a bootstrap approach to compare the predicted 3/5/10-year OS with the observed OS. All resampling times were set to 1,000. The calibration curve was along the 45° line of the calibration plot in the perfect calibration model, indicating that the predicted OS probabilities were identical to the actual probabilities. A decision curve analysis (DCA) was used to assess the actual benefits for patients.

All statistical analyses were performed using SPSS 23.0 (Armonk, NY: IBM Corp.) and R 4.2.2 (http://www.r-project.org/). *p*-value < 0.05 was defined as meaningful and all statistical tests were two-sided.

## Results

### Clinical characteristics of elderly patients with breast cancer

A total of 2,231 elderly patients with breast cancer were enrolled in our cohort from the PUMCH, and the median age of the patients was 71.0 years (IQR, 67–76). Patients aged 65–69, >70 years, and >80 accounted for 37.1%, 62.9%, and 12.6%, respectively. Patients with luminal, HER2, and triple-negative breast cancers (TNBC) accounted for 79.7%, 9.1%, and 11.2% of the patients, respectively. The largest proportion of patients were in TNM stage I (57.7%). The CCI (0), CCI (1), CCI (2), and CCI (3) values were 60.3%, 24.5%, 10.2%, and 5.0%, respectively. The patients were randomly divided into a training cohort (*n* = 1,515) and a validation cohort (*n* = 716). Their demographics and clinical features are listed in **Table 1**. The baseline characteristics and outcome data were well balanced between the two cohorts.

**Table 1:** Demographic and clinicopathologic variables of 2,331 elderly patients with breast cancer in the training and validation cohort.

	Training Cohort	Validation Cohort	*p*
	*n* = 1515	*n* = 716	
Age median (IQR), years	71 (67.0–76)	71 (67–76)	0.855
T stage			0.971
1	1,002 (66.1%)	470 (65.6%)	
2	481 (31.7%)	231 (32.3%)	
3	32 (2.11%)	15 (2.09%)	
Tumor differentiation			0.048
Well	255 (16.8%)	132 (18.4%)	
Moderately	895 (59.1%)	384 (53.6%)	
Poorly	365 (24.1%)	200 (27.9%)	
Lymph node dissection			0.580
No	1,161 (76.6%)	557 (77.8%)	
Yes	354 (23.4%)	159 (22.2%)	
N stage			0.809
0	1,165 (76.9%)	558 (77.9%)	
1	184 (12.1%)	80 (11.2%)	
2	77 (5.08%)	40 (5.59%)	
3	89 (5.87%)	38 (5.31%)	
TNM stage			0.859
1	879 (58.0%)	409 (57.1%)	
2	465 (30.7%)	228 (31.8%)	
3	171 (11.3%)	79 (11.0%)	
HR			0.302
Negative	302 (19.9%)	157 (21.9%)	
Positive	1,213 (80.1%)	559 (78.1%)	
HER2			0.534
Negative	1,012 (66.8%)	468 (65.4%)	
Positive	503 (33.2%)	248 (34.6%)	
Ki67			0.699
≤14%	624 (41.2%)	288 (40.2%)	
>14%	891 (58.8%)	428 (59.8%)	
Pathological type			0.394
Luminal	1,218 (80.4%)	559 (78.1%)	
HER2	131 (8.65%)	73 (10.2%)	
TNBC	166 (11.0%)	84 (11.7%)	
CCI status			0.675
0	910 (60.1%)	436 (60.9%)	
1	368 (24.3%)	179 (25.0%)	
2	156 (10.3%)	71 (9.92%)	
3	81 (5.35%)	30 (4.19%)	
Operation type			0.642
1	571 (37.7%)	274 (38.3%)	
2	177 (11.7%)	74 (10.3%)	
3	767 (50.6%)	368 (51.4%)	
Chemotherapy			0.330
No	923 (60.9%)	420 (58.7%)	
Yes	592 (39.1%)	296 (41.3%)	
Radiotherapy			0.503
No	1,242 (82.0%)	596 (83.2%)	
Yes	273 (18.0%)	120 (16.8%)	
Hormone therapy			0.823
No	318 (21.0%)	154 (21.5%)	
Yes	1,197 (79.0%)	562 (78.5%)	
Targeted therapy			0.740
No	1,402 (92.5%)	659 (92.0%)	
Yes	113 (7.46%)	57 (7.96%)	
Outcomes			0.493
Survival	1,350 (89.1%)	631 (88.1%)	
Death	165 (10.9%)	85 (11.9%)	

### Characteristics of elderly patients with breast cancer significantly related to OS

In all 2,231 patients, the 5- and 10-year OS was 91.3% and 78.4%, respectively. The Kaplan–Meier survival curve was used to analyze the relationship between clinical and pathological features and OS. Patients with different CCI statuses, T stages, N stages, and TNM stages had significantly different OS (*p* = 0.006, *p* < 0.001, *p* < 0.001, *p* < 0.001, **Supplementary Figures 1A–D**). Patients with better tumor differentiation (well/moderately) had significantly higher OS than patients with poor differentiation (*p* < 0.001, **Supplementary Figure 1E**). Patients with KI-67 ≤ 14% had significantly higher OS than the patients with KI-67 > 14% (*p* < 0.001, **Supplementary Figure 1G**). Luminal patients had significantly higher OS than HER2 and TNBC patients (*p* = 0.033, **Supplementary Figure 1H**). In terms of treatment, patients who had undergone different operation types had significantly different OS (*p* < 0.001, **Supplementary Figure 1I**). Patients who had undergone radiotherapy had significantly higher OS than patients who did not receive radiotherapy (*p* = 0.006, **Supplementary Figure 1J**). Patients who received endocrine therapy had significantly higher OS than patients who did not receive endocrine therapy (*p* = 0.003, **Supplementary Figure 1K**). However, patients who received chemotherapy had lower OS than patients who did not receive chemotherapy (*p* = 0.004, **Supplementary Figure 1L**).

### Univariate Cox and multivariate Cox analyses of the OS

A univariate Cox regression model was used to explore the relationships between the 16 predictive variables listed in **Table 1** and OS. Eleven candidate variables, such as age, clinicopathological variables, and adjuvant therapy, were significantly associated (*p* < 0.05) with OS in univariate analysis and were further included in multivariate analysis. Backward stepwise selection using AIC in Cox proportional hazards regression modeling identified the following eight variables that had the strongest association with the OS of patients with breast cancer in our training cohort: age, T stage, N stage, Ki-67, CCI status, operation type, radiotherapy, and endocrine therapy (**Table 2**).

**Table 2:** Prognostic factors identified by univariate and multivariate Cox regression analyses in the training cohort.

	Univariate analysis				Multivariate analysis		
	HR	95% CI	*p*		HR	95% CI	*p*
Age, years	1.132	1.103–1.161	<0.001		1.123	1.088–1.158	<0.001
T stage							
1	1				1		
2	1.551	1.129–2.129	0.007		1.293	0.912–1.832	0.149
3	3.896	1.887–8.043	<0.001		4.048	1.895–8.647	<0.001
Tumor differentiation							
Well	1						
Moderately	0.943	0.625–1.422	0.778				
Poorly	1.782	1.157–2.746	0.009				
Lymph node dissection							
No	1						
Yes	1.292	0.904–1.846	0.160				
N stage							
0	1				1		
1	0.726	0.401–1.314	0.260		1.081	0.562–2.082	0.815
2	1.281	0.691–2.373	0.432		2.067	1.043–4.098	0.037
3	3.234	1.932–5.413	<0.001		5.562	3.050–10.144	<0.001
TNM stage							
1	1						
2	1.307	0.927–1.844	0.127				
3	2.357	1.542–3.603	<0.001				
HR							
No	1						
Yes	0.708	0.495–1.013	0.059				
HER2							
No	1						
Yes	1.312	0.924–1.864	0.129				
Ki67							
≤14%	1				1		
>14%	1.741	1.264–2.398	0.001		1.801	1.284–2.527	0.001
Pathological type							
Luminal	1						
HER2	1.425	0.804–2.528	0.225				
TNBC	1.355	0.882–2.080	0.166				
CCI status							
0	1				1		
1	1.442	0.998–2.084	0.051		1.272	0.876–1.848	0.206
2	1.600	1.017–2.518	0.042		1.428	0.901–2.262	0.129
3	2.232	1.347–3.698	0.002		2.394	1.425–4.023	0.001
Operation type							
1	1				1		
2	0.333	0.135–0.821	0.017		0.733	0.281–1.908	0.524
3	0.548	0.399–0.753	<0.001		0.583	0.384–0.885	0.011
Chemotherapy							
No	1						
Yes	1.566	1.149–2.136	0.005				
Radiotherapy							
No	1				1		
Yes	0.358	0.167–0.767	0.008		0.402	0.178–0.910	0.029
Endocrine therapy							
No	1				1		
Yes	0.595	0.423–0.836	0.003		0.610	0.429–0.866	0.006
Targeted therapy							
No	1						
Yes	0.712	0.291–1.743	0.457				

### Construction of predictive nomograms for OS

**Figure 1:**
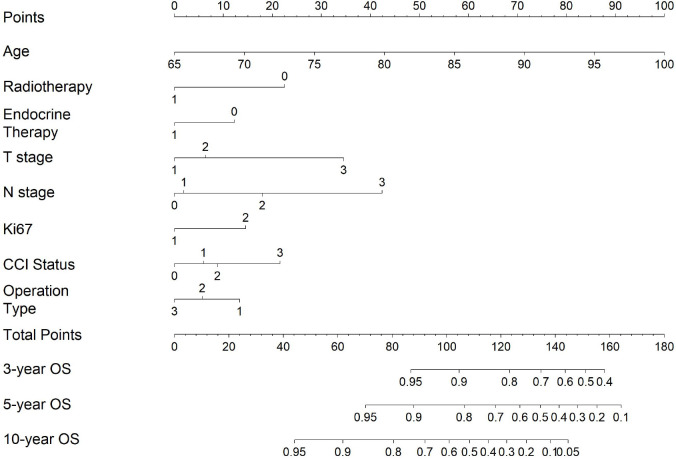
A prognostic nomogram for predicting OS in elderly patients with breast cancer.

We constructed a predictive nomogram based on the Cox proportional hazards model (**Figure 1**). Each predictor variable was assigned a score and read out on the top scale. By summing the scores and locating them on the total score scale, the estimated probability of 3-, 5-, and 10-year OS was determined (**Figure 1**), and higher total scores were associated with worse prognosis.

### Validation of predictive nomogram for OS

Since our prediction model was built using two sets obtained through a random split, in order to avoid inappropriate variables being included in the model due to this contingent grouping method, we utilized bootstrap technology to validate the model’s discrimination ability and enhance its robustness (23, 24). The resampling times were set to 1,000. Bootstrap is a flexible and powerful statistical tool that relies on random sampling with replacement and can be used in some situations such as validation of predictive model performance. In our training cohort, we obtained 1,000 subsets using bootstrap resampling 1,000 times, and constructed 1,000 models accordingly. The average C-index of these models represents the discriminative ability of the model, so it can mitigate the possibility of a model with a high discriminatory power only in a specific population caused by contingency. The bootstrap C-index was 0.798 (95% CI, 0.764–0.831) in the training cohort. It indicated that the model had good discrimination in the training cohort. We replicated the above procedure to assess the model’s discriminative ability in the validation cohort, yielding a C-index of 0.793 (95% CI, 0.736–0.842). The 95% confidence interval experienced a slight increase, but the results were closely aligned with those of the training group. These results showed that our predictive model had good discrimination.

The similarity between survival probabilities predicted by the nomogram and actual survival rates was assessed with calibration plots; to avoid contingency, we use the bootstrap method to present the calibration plot and set resampling times to 1,000.

**Figure 2:**
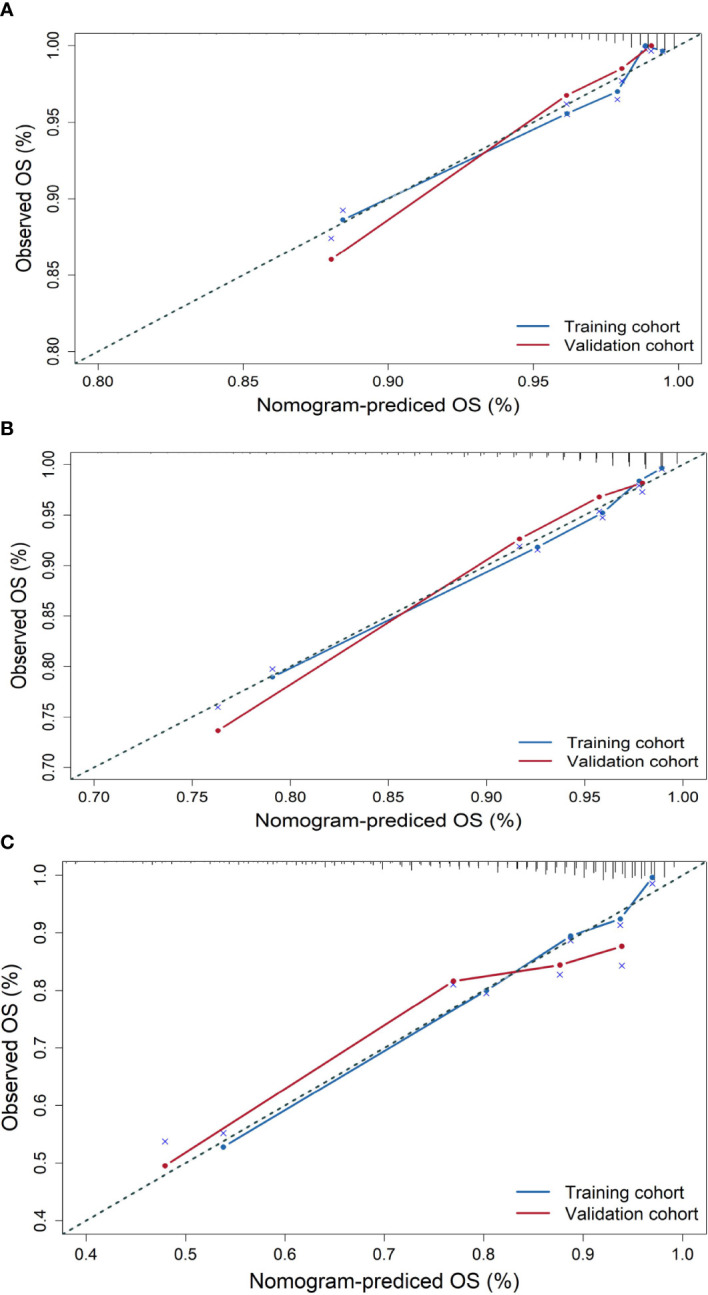
Calibration plots for the prediction of OS for elderly patients with breast cancer at 3, 5, and 10 years in the training cohort and validation cohort. **(A)** Calibration plots for 3-year OS, **(B)** calibration plots for 5-year OS, and **(C)** calibration plots for 10-year OS.

The similarity between survival probabilities predicted by the nomogram and actual survival rates was assessed with calibration plots. We used the bootstrap method to present the calibration plot and set resampling times to 1,000 to avoid contingency. The results showed that the actual 3-, 5-, and 10-year survival rates corresponded closely to the predicted survival probabilities in the validation cohort (**Figure 2**).

**Figure 3:**
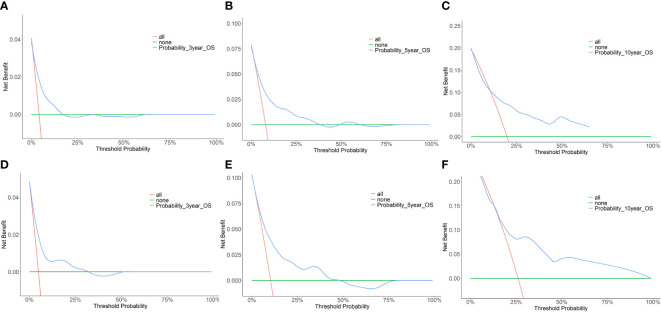
Decision curve analysis (DCA) of the nomogram predicting 3-year, 5-year, and 10-year OS for the elderly patients with breast cancer. **(A)** DCA for 3-year OS in the training cohort. **(B)** DCA for 5-year OS in the training cohort. **(C)** DCA for 10-year OS in the training cohort. **(D)** DCA for 3-year OS in the validation cohort. **(E)** DCA for 5-year OS in the validation cohort. **(F)** DCA for 10-year OS in the validation cohort.

Furthermore, the DCA for the nomograms of 3-, 5-, and 10-year OS was presented. **Figure 3** shows the results of the DCA of the nomogram in the training cohorts **(A–C)**, and in the validation cohorts **(D–F)** for 3-, 5-, and 10-year OS. In the DCA plot, the blue line represents the net benefit based on the nomogram predictive model. The red and green lines represent the net benefits of the strategy for treating all and no patients, respectively. DCA demonstrated that our nomogram model was superior to both all-treatment and no-treatment regimens in predicting the survival of elderly patients with breast cancer. From the DCA curves, our nomogram was able to better predict the 3-, 5-, and 10-year OS in both the training and validation cohorts.

**Figure 4:**
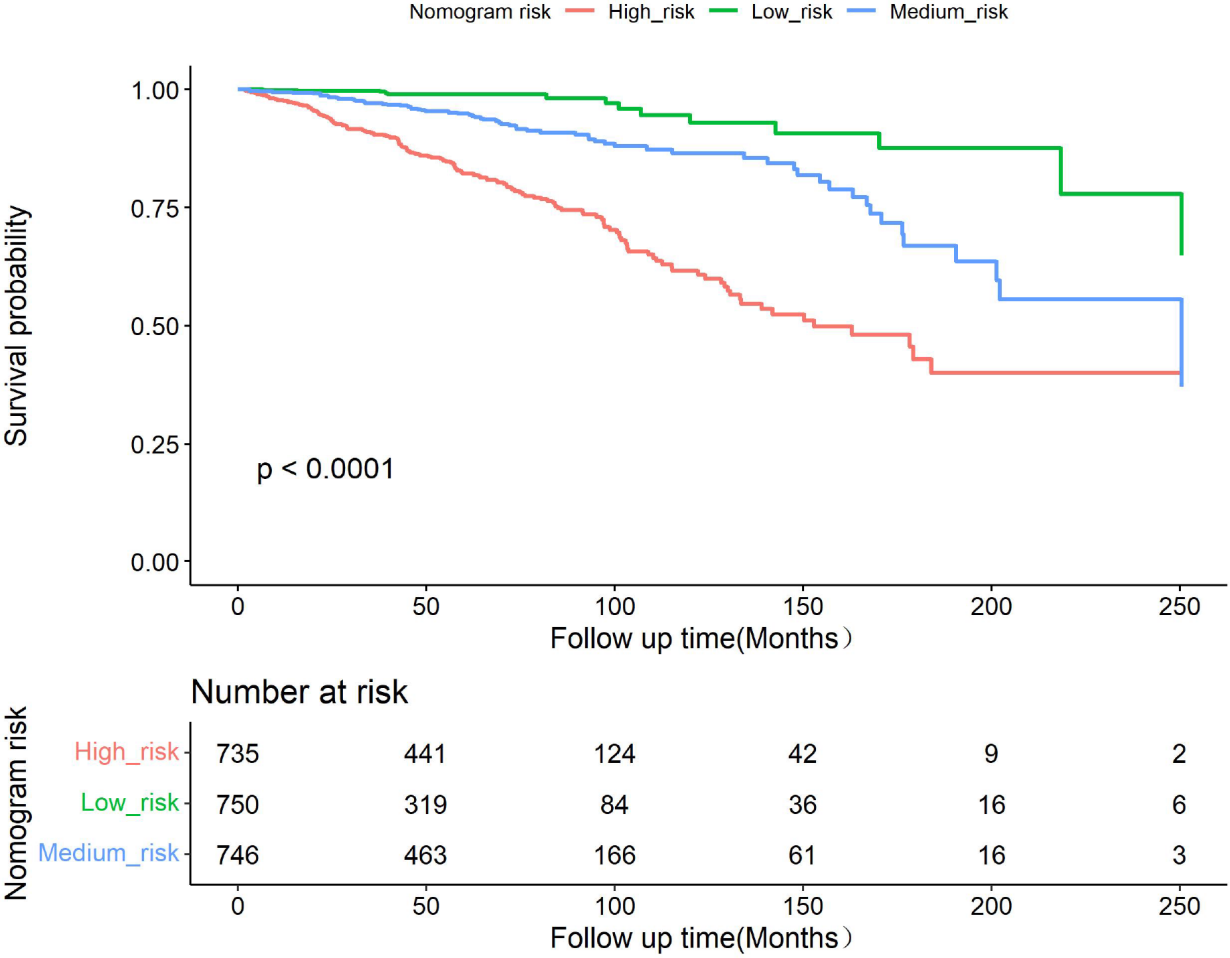
Kaplan–Meier curves showing survival in elderly patients with breast cancer according to predicted OS tertiles.

To further evaluate the discriminative ability of the model, the predicted probability of OS was compared using the log-rank test and plotted as Kaplan–Meier curves stratified by the tertile of the predicted probability calculated from the nomogram. The patients were divided into three risk groups: low risk (total points < 78), medium risk (78 ≤ total points < 108), and high risk (total points ≥108). Kaplan–Meier OS curves showed significant differences among the three risk groups (**Figure 4**).

## Discussion

This analysis identified CCI and seven clinicopathological parameters and treatment methods as strong predictors for OS in patients with breast cancer that were ≥65 years old. This study showed that the predictive nomogram can accurately predict 3-, 5-, and 10-year OS in patients with breast cancer ≥65 years old. Because competing mortality risks, including comorbidities and aging, are more prevalent in elderly adults, treatment decisions should consider not only the risk of breast cancer recurrence but also the risk of dying from comorbidities (25). Our predictive nomogram not only accurately evaluated OS, but also included the comorbidity indicator CCI, which competes with the causes of mortality in elderly patients (26). Adjuvant! Online is the most widely used predictive model for breast cancer patients (12) and can predict 10-year OS and provide the expected benefits of chemotherapy and endocrine therapy (27); however, the maximum age of patients in the cohort was 69 years, and it does not accurately predict OS in elderly patients (13, 27, 28). Our predictive nomogram, called PEEBC, was constructed using a large cohort of 2,231 patients aged ≥65 years, with a median age of 71 years and 63.9% of patients >70 years of age. As a predictive model specifically for elderly patients with breast cancer, it is more accurate in elderly patients. PREDICT, a predictive model for elderly patients with breast cancer, performed better because it was based on a large cohort. However, it overestimated the 10-year OS and did not include comorbidities (14). There are other gene score-based predictive models, including Oncotype DX and MammaPrint; however, these models have not been adequately validated in the elderly breast cancer population, as most of the patients recruited in these studies were under 65 years old (29, 30). Recently, there have been some novel prognostic models for elderly patients with breast cancer (31, 32); however, the data for these nomograms from public databases were heterogeneous and the model was applicable to specialized groups such as patients with TNBC (31) or metastasis (32). The predictive model constructed in our study included not only the main clinicopathological features such as T stage, N stage, KI-67 status, and primary treatment methods such as operation type, but also chemotherapy and radiotherapy. The nomogram included not only tumor load but also age and CCI score, which are competing mortality risks in elderly patients. In our study, using continuous patients from the same hospital avoided bias in the pathological diagnosis and comorbidity judgment. Unlike other prediction models, the type of operation was included as an important factor in our predictive nomogram (12, 14). Although standard surgical treatment remains in use in some elderly patients with early disease, there is a risk of overtreatment in patients with competing mortality risks (6). Surgery with tamoxifen was shown to achieve better local control than tamoxifen alone (33). Older patients with breast cancer with clinically negative nodes do not benefit from immediate axillary dissection in terms of breast cancer mortality (34). Therefore, sentinel lymph node surgery may be omitted in elderly women at low risk of nodal positivity (35). Thus, the type of operation is tailored to the age and comorbidities of elderly patients with breast cancer.

The Kaplan–Meier curve showed that T stage, N status, and Ki-67 expression in elderly patients were significantly associated with OS, which is consistent with the findings in other breast cancer patients. Patients with high CCI scores had a lower OS than those with low CCI scores. Notably, patients who received chemotherapy had a lower OS than those who did not. In previous studies, the benefits of chemotherapy for survival in elderly patients were observed only in groups with ER− cancer (36, 37). Another cohort study found that in node-positive, ER+ elderly patients with breast cancer with multiple comorbidities, receiving chemotherapy was associated with improved OS (38). In our cohort, older patients did not benefit from chemotherapy or had worse survival because the most common chemotherapy regimen was capecitabine, fewer patients received intravenous chemotherapy, and most patients were HR+. The heterogeneity of elderly patients was high; therefore, the benefit of chemotherapy in elderly patients requires further study.

The PRIME II study found that postoperative whole-breast radiotherapy did not affect the OS of elderly patients with HR+ and lymph node (LN)− cancer (39, 40). In our cohort, radiotherapy was included in the nomogram prediction, and patients who received radiotherapy had a higher OS than patients who did not receive radiotherapy. A possible reason for the survival benefit from radiotherapy is that LN+ patients accounted for 23.3%, T2 and T3 patients accounted for 34.1%, and the tumor load was relatively high in our cohort. Therefore, radiotherapy plays an important role in the treatment of elderly patients with breast cancer.

The prevalence of breast cancer among elderly patients is increasing, and cancer-related mortality is higher in elderly adults than in younger women (41). Elderly patients with TNBC have a poorer prognosis than younger patients with TNBC (42). Most patients included in clinical research on breast cancer are patients under 70 years of age, and few clinical studies have focused on elderly patients with breast cancer. To improve the survival of elderly patients with breast cancer, we should not only consider the characteristics of the tumor but also the comorbidities of the patients, their physical function, and treatment willingness.

This was a single-center study, and patients who visited our center may have certain clinical characteristics, which may have led to a potential selection bias. The study population was randomly divided into the training and validation cohorts. This kind of validation is not an external validation in the strict sense, but a kind of internal validation that cannot completely avoid overfitting. This limits extrapolation of the prediction model.

The predictive nomogram of PEEBC that we have established is consistent with the real-world situation of elderly patients with breast cancer and is a starting point for the future evaluation of elderly patients with breast cancer. In addition to adequate antitumor treatment, the expected survival of elderly patients with breast cancer should be assessed using a nomogram to obtain a tailored treatment plan, prolong survival time, and improve the quality of life.

## Data availability statement

The raw data supporting the conclusions of this article will be made available by the authors, without undue reservation.

## Ethics statement

The studies involving human participants were reviewed and approved by the Institutional Reviewer Board of the Peking Union Medical College Hospital. The patients/participants provided their written informed consent to participate in this study.

## Author contributions

YZ, YDZ, YX, ZW, FM, SS, YL, and QS conducted the study and collected the data. YZ and KS analyzed the data and interpreted the results. All authors contributed to the article and approved the submitted version.
