# Supplementary figures and images for: A nomogram for individually predicting overall survival for elderly patients with early breast cancer: a consecutive cohort study
Source: Front Oncol. 2023 Jul 28;13:1189551. doi: 10.3389/fonc.2023.1189551 (PMC10420132; doi:10.3389/fonc.2023.1189551)

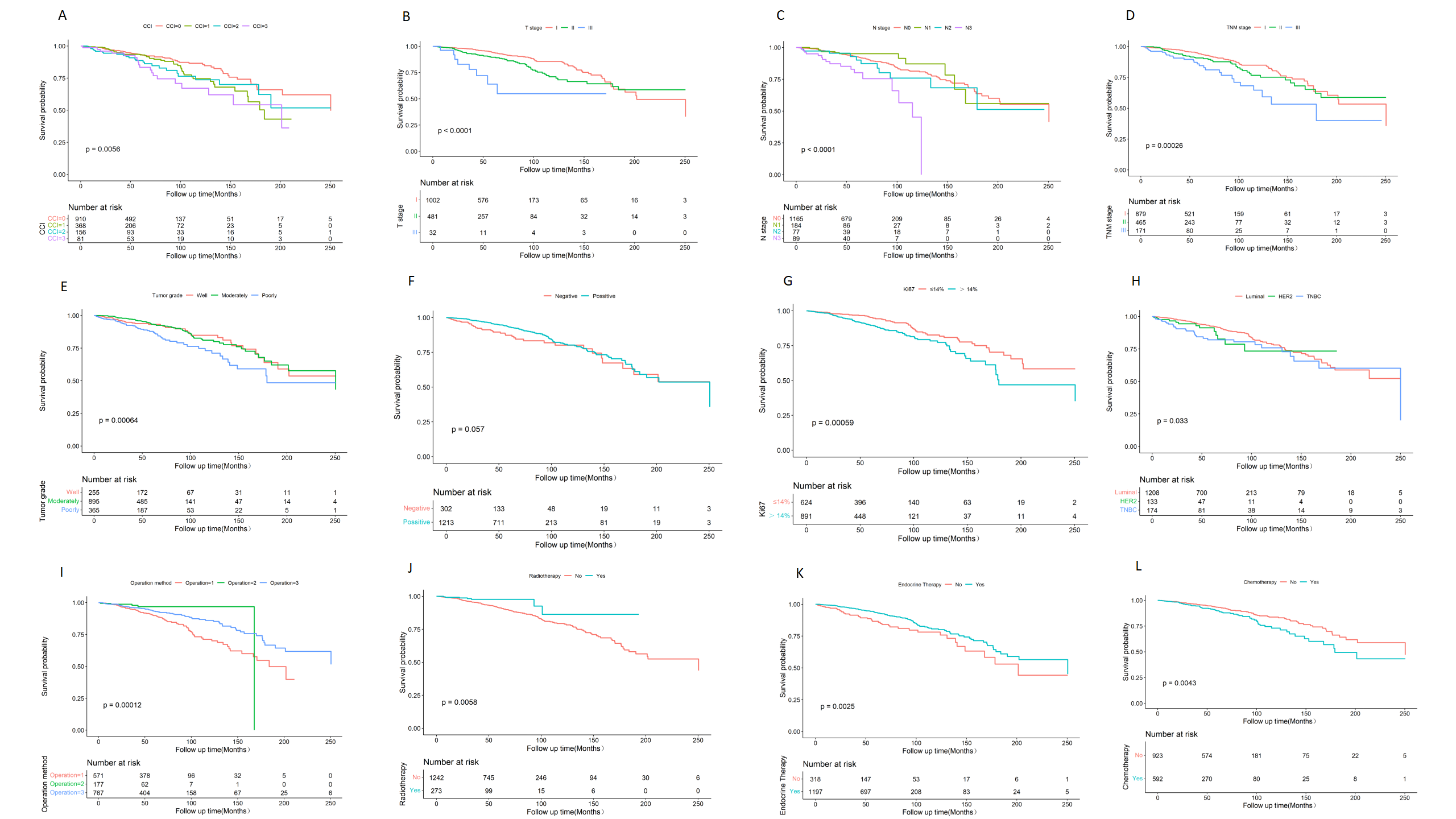

Supplement: Supplementary file 1 [file Image_1.tif]
